# Supplementary material for: Preexisting risk-avoidance and enhanced alcohol relief are driven by imbalance of the striatal dopamine receptors in mice
Source: Nat Commun. 2024 Oct 22;15:9093. doi: 10.1038/s41467-024-53414-y (PMC11496688; doi:10.1038/s41467-024-53414-y)
Supplement: Supplementary file 1 — Supplementary Information [file 41467_2024_53414_MOESM1_ESM.pdf]

Supplemental Material and Figures

Preexisting risk-avoidance and enhanced alcohol relief are driven by imbalance in striatal dopamine receptors in mice

Miriam E. Bocarsly*[1,2], Marlisa J. Shaw[1,3], Emilya Ventriglia[4], Lucy G. Anderson[5], Hannah C. Goldbach[1,5], Catherine E. Teresi[1,6], Marilyn Bravo[1], Roland Bock[1,5], Patrick Hong[2], Han Bin Kwon[1], Imran M. Khawaja[2], Rishi Raman[2], Erin M. Murray[1], Jordi Bonaventura[4,7], Dennis A. Burke[1], Michael Michaelides[4], and Veronica A. Alvarez*[1,4,5,6]

1. Laboratory on Neurobiology of Compulsive Behaviors, National Institute on Alcohol Abuse and Alcoholism, Intramural Research Program, NIH, Bethesda, MD, USA
2. Department of Pharmacology, Physiology and Neuroscience, Brain Health Institute, Rutgers New Jersey Medical School, Newark, NJ, USA
3. NIH Academy Enrichment Program, Office of OITE
4. National Institute on Drug Abuse, Intramural Research Program, NIH, Baltimore, MD, USA
5. National Institute on Mental Health, NIH, Bethesda, MD, USA
6. Center on Compulsive Behaviors, NIH, Bethesda, MD, USA
7. Institut de Neurociències, Universitat de Barcelona, Barcelona, Spain

*, these authors jointly supervised this work; correspondence to be addressed to Miriam Bocarsly (bocarsme@njms.rutgers.edu) and Veronica Alvarez (alvarezva@mail.nih.gov)

Suppl Fig 1 Bocarsly et al.

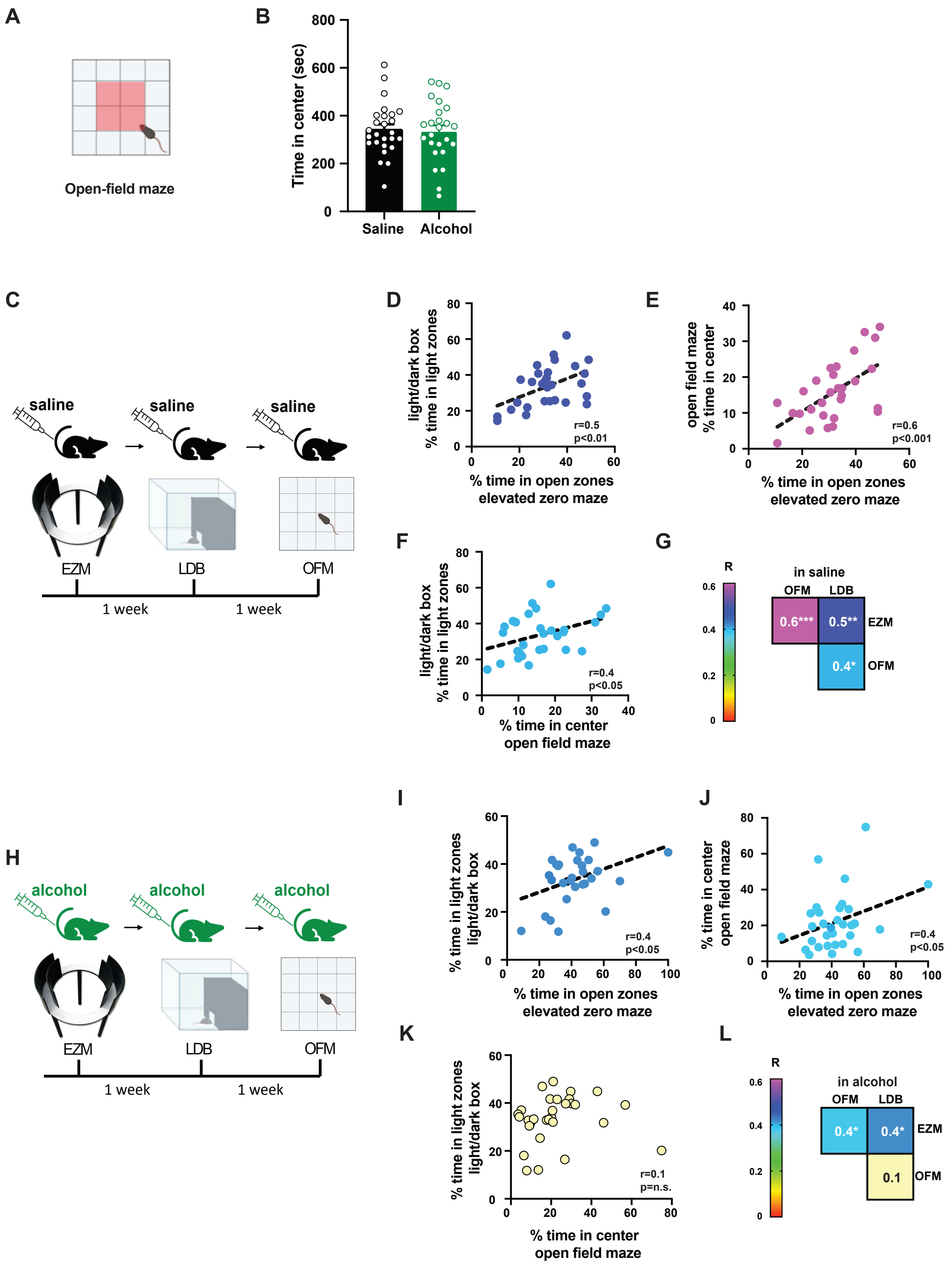

Supplemental Figure 1.

(A) Mice were injected with either saline or alcohol (1.2 g/kg, i.p.) and allowed to explore an open field. (B) Consistent with previous findings, mice did not spend more time in the center of the open-field maze when treated with alcohol compared to saline ($t(48)=0.3$, $p>0.05$, n=25-26/group). (C) Mice were tested across a variety of behavioral risk-avoidance tasks following administration of saline or alcohol (1.2 g/kg, i.p.). (D-G) While mice showed individual variation on behavioral exploration tasks, correlations were noted between individual animal performance across the tasks following saline. (D: LD vs. EZM: $F(1,27)=8.02$, $p<0.01$; E: OFM vs. EZM: $F(1,27)=14.86$, $p<0.001$; F: LD vs. OFM: $F(1,27)=4.65$, $p<0.05$, G: summary statistics, n=29 mice). (H) Similar correlations were also observed on the behavioral risk-avoidance tasks following administration of alcohol (1.2 g/kg) except that the percent time in the center of the open-field maze did not correlate with the other behavioral tasks following alcohol. (I: LD vs. EZM: $F(1,29)=5.73$, $p<0.05$; J: OFM vs. EZM: $F(1,29)=4.11$, $p=0.05$; K: LD vs. OFM: $F(1,29)=0.40$, $p>0.05$, L: summary statistics, n=31). Data presented as mean ± SEM, *$p<0.05$, **$p<0.01$, ***$p<0.001$.

Suppl Fig 2 Bocarsly et al.

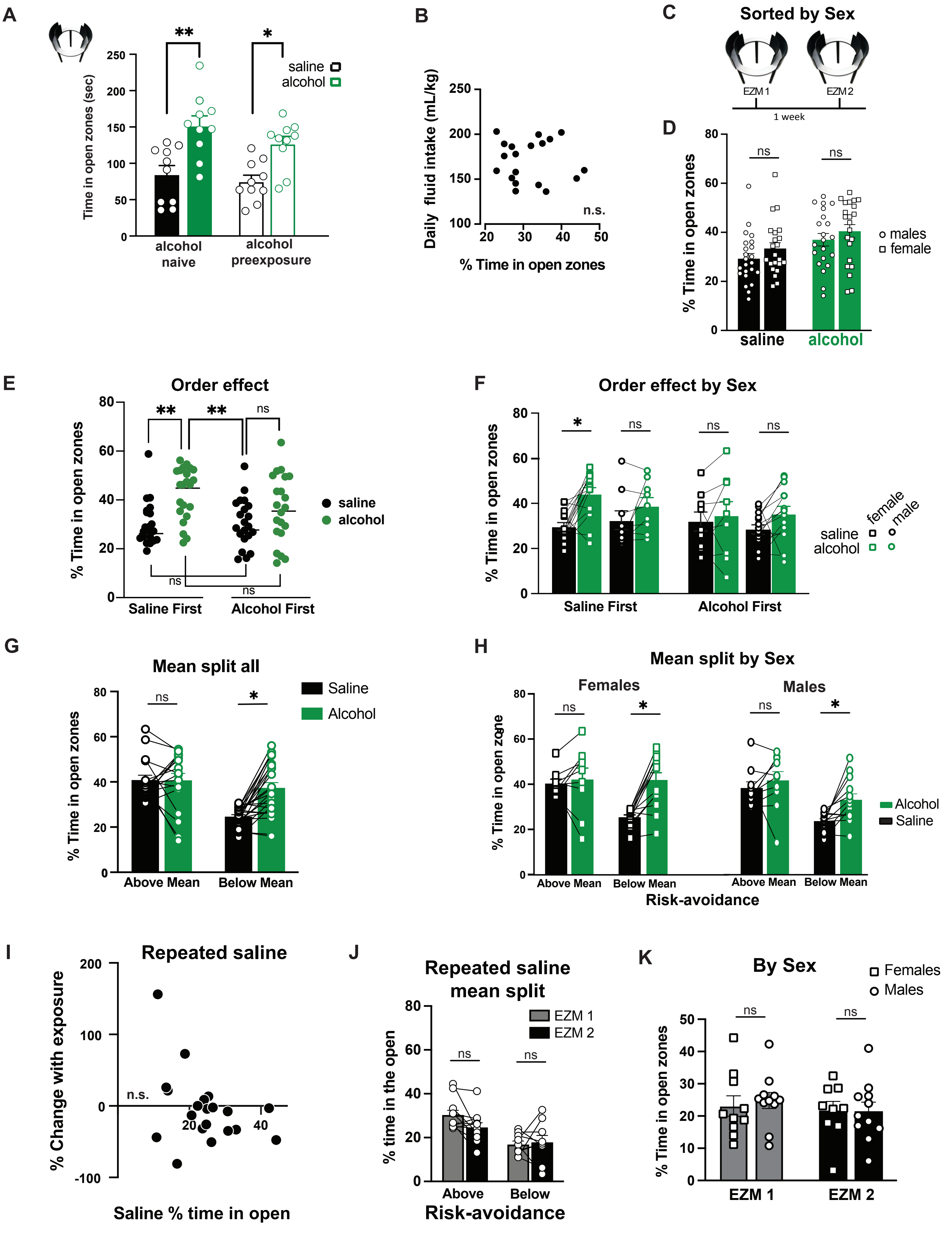

Supplemental Figure 2.

(A) Previous alcohol exposure did not affect the outcomes on the EZM performance (interaction effect, $F(3,36)=9.88$, $p<0.0001$, no main effect of past alcohol exposure $p>0.05$, n=10/group). Mice receiving alcohol (1.2 g/kg, i.p.) for the first time or after repeated pre-exposure in the home cage showed similar increase in time spent on EZM open zones (p's<0.01). **(B)** A cohort of mice were tested on the EZM following alcohol administration (1.2 g/kg, i.p.), and then allowed to consume water and alcohol using an intermittent-access two-bottle test. The total volume consumed was not correlated with time spent in EZM open zones following alcohol ($F(1,17) = 0.08$ $p>0.05$, n=19). **(C-D)** Data from repeated EZM experiment shown main Figure 1J-K is sorted by sex. Mice were run on the EZM twice one week apart and they received either saline or alcohol, in a counterbalanced design. No statistical differences were noted between males and females on time spent on the open arm of the repeated EZM following saline or alcohol (sex x drug interaction: $F(1,46) = 1.16$ $p>0.05$, n=45). **(E-F)** Same data as Figure 1JK was sorted by order of drug administered to determine possible effects of the counterbalanced design. In panel E, analysis shows both sexes combined with no significant interaction and a limited effect of the order (drug x order interaction: $F(1,41) = 3.45$ $p>0.05$). In panel F, data is shown further sorted by sex in male (circle) and females (square) showing a significant effect of alcohol ($F(1, 14) = 8.046$ $p<0.02$) and significant interaction between alcohol and order/Sex ($F(3,16) = 3.4$ $p=0.04$). Likely due to the smaller sample size, the only significant comparison between saline and alcohol was in the group of female mice that received saline first. **(G)** Same data as Figure 1L, is now sorted by mean time spent in EZM open zones after saline. Only mice below mean (high risk avoidance) group, show significantly increased time in the open zones of the EZM following alcohol compared to saline (risk avoidance x drug interaction effect: $F(1,41) = 11.11$ $p<0.01$; main effect of drug: $F(1,41) = 10.52$ $p<0.01$; main effect of risk avoidance: $F(1,41) = 17$ $p<0.001$). **(H)** Above and below mean analysis of the same data by sex shows significant alcohol effect only in the "below mean" group for both female ($p<0.0001$) and male ($p<0.01$) mice (females: significant effect of Alcohol, Mean-split and interaction AlcoholxMean-split $Fs(1,20) > 4.5$, ps <0.04, n=22; males: significant effect of Alcohol and Mean-split $F(1,21)>7.5$ ps<0.01, n=23).

(I) Experimental validation of repeated EZM tests done with repeated saline administrations shows the percent change in time spent in open zones after the first and second saline test ($F(1,18)=3.09$, $p>0.05$; n=20). **(J)** Median split of first saline EZM performance into high (below mean) and low (above mean) risk-aversion groups shows no differences in time spent on EZM open zones during the first and second saline tests ($F(1,18)=2.97$, $p>0.05$, n=20). *p<0.05, **p<0.01. **(K)** Repeated EZM experiment with saline administration is shown sorted by sex. No significant sex differences nor differences between first and second EZM test were found ($F(1,37) < 0.69$, ps > 0.4, n=41). For all panels, bars represent mean ± SEM and symbols show the individual mice data.

Suppl Fig 3 Bocarsly et al.

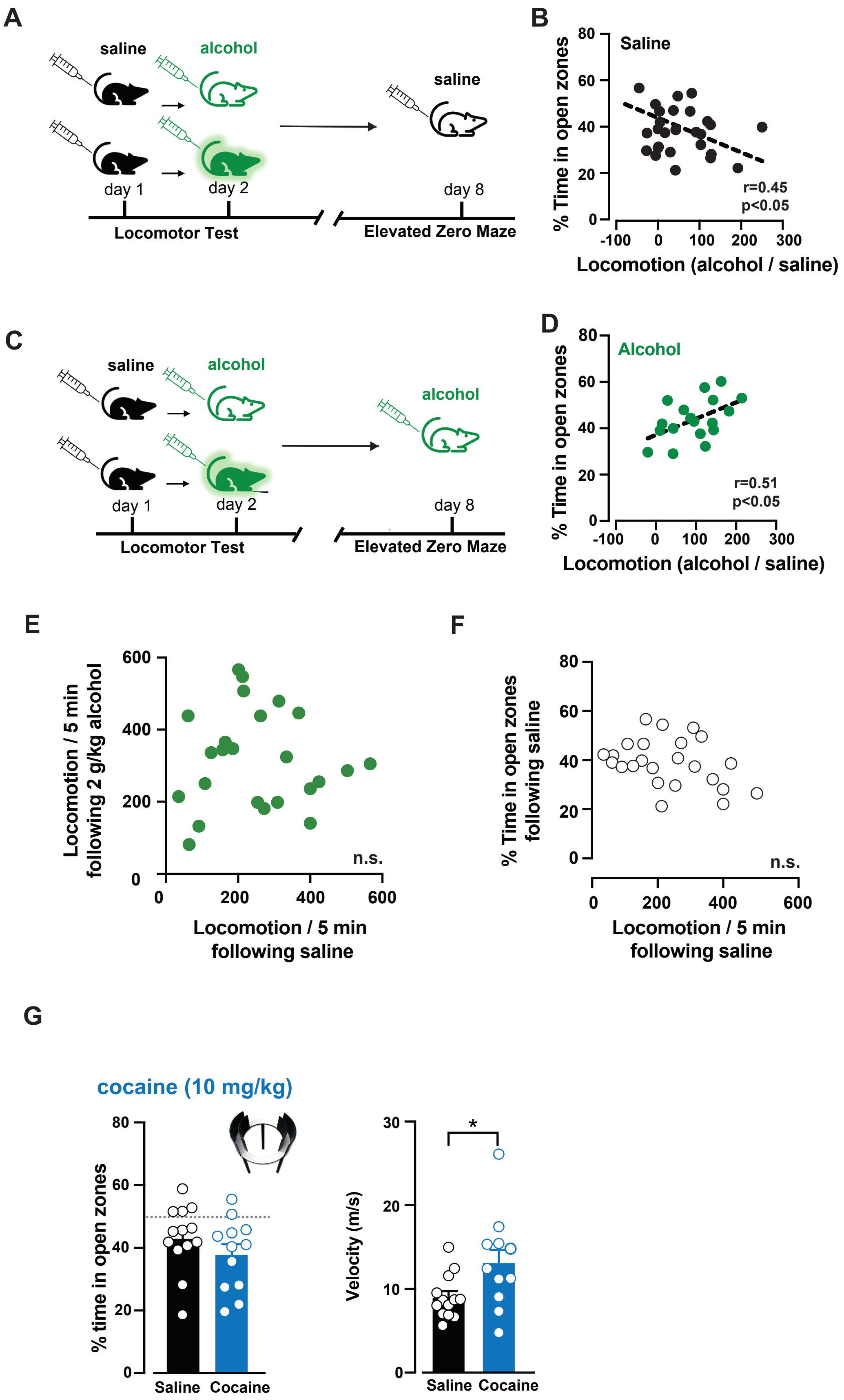

Supplemental Figure 3.

(A,C) Experimental design shows repeated locomotor tests were performed after saline and 2 g/kg alcohol and one week later mice were tested in the EZM after saline (A) or 1.2 g/kg alcohol (C). (B) An inverse relationship was found between alcohol-induced locomotion (alcohol/saline) and the time spent on EZM open zones after saline ($F(1,26)=4.45$, $p<0.05$, n=27). (D) A positive correlation was found between alcohol-induced locomotion (alcohol/saline) and the time spent on EZM open zones after alcohol ($F(1,16)=5.63$, $p<0.05$, n=17. (E) No correlation between locomotion response after saline vs. after alcohol ($F(1,22)=0.00004$, $p>0.05$, n=23). (F) No correlation between locomotion after saline and time spent on EZM open zones after saline ($F(1,22)=3.03$, $p>0.05$, n=23). (G) Left, No difference in time spent on EZM open zones after cocaine (10 mg/kg, i.p.) compared to after saline (left; $t(22)=1.0$, $p>0.05$, n=13, 12 mice). Right, same cocaine dose produced locomotor stimulation (right; $t(22)=2.37$, $p<0.05$, n=13-12). Data presented as mean ± SEM, *$p<0.05$.

Suppl Fig 4 Bocarsly et al.

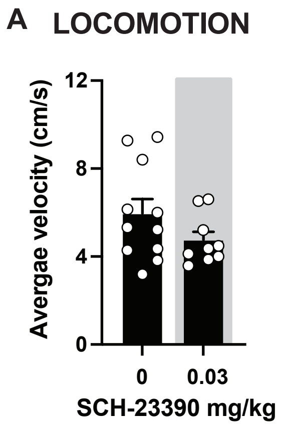

Supplemental Figure 4.
Control mice showed no differences in movement velocity following administration of the D1-like antagonist SCH-23390 (0.03 mg/kg) as compared to saline ($t(18)=1.58$, $p>0.05$, n=9-11/group). Data presented as mean ± SEM.

Suppl Fig 5 Bocarsly et al.

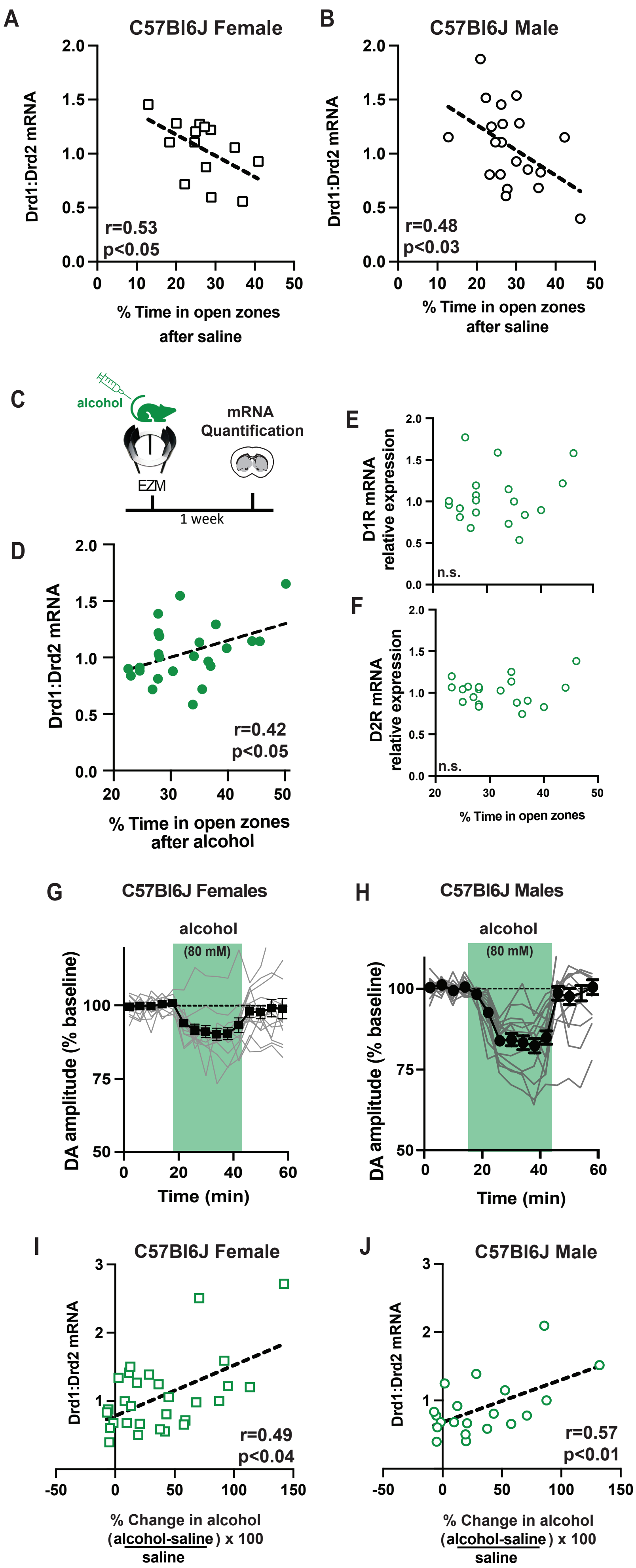

Supplemental Figure 5.

(A-B) Data from Figure 3A is sorted by sex showing a similar negative correlation between the EZM performance after saline and the ratio of Drd1 to Drd2 mRNA in dorsomedial striatal samples of female ($F(1,12)=4.75$, $p<0.05$, $n=14$) and male mice ($F(1,19)=5.25$, $p<0.05$, $n=21$).
(C) Mice were allowed to explore the EZM following alcohol administration (1.2 g/kg, i.p.), and then tissue was taken for analysis of Drd1 and Drd2 mRNA in the dorsal striatum using qPCR.
(D) A positive correlation was found between EZM performance after alcohol and the ratio of Drd1 to Drd2 mRNA expression in dorsomedial striatal samples ($F(1,22)=4.80$, $p<0.05$, $n=24$).
(E-F) Neither Drd1 mRNA levels nor Drd2 mRNA levels were correlated with the behavior when measured independently (E: $F(1,22)=0.62$, $p>0.05$ and F: $F(1,22)=0.02$, $p>0.05$, $n=24$).
(G-H) Alcohol effect on in vitro measurements of evoked dopamine signals in the dorsomedial striatum using fast-scan cyclic voltammetry. Female and male mice were first screened on their EZM performance and later brain tissue was taken for in vitro analysis. Bath application of 80 mM alcohol reduced the amplitude of the evoked dopamine signals in the dorsomedial striatum of both females and males but alcohol effect was smaller in females (main effect of alcohol $F(14,420)= 31.67$, $p>0.0001$; main interaction alcohol x sex $F(14,420)=3.5$, $p>0.0001$, $n=32$). Normalized data from individual brain slices are shown in gray lines and the mean and SEM in symbols and bars.
(I-J) Data from Figure 3C is sorted by sex showing a similar positive correlation between the EZM performance after alcohol and the ratio of Drd1 to Drd2 mRNA in dorsomedial striatal samples of female ($F(1,15)=4.83$, $p<0.04$, $n=17$) and male mice ($F(1,18)=8.57$, $p<0.01$, $n=20$).

Suppl Fig 6 Bocarsly et al.

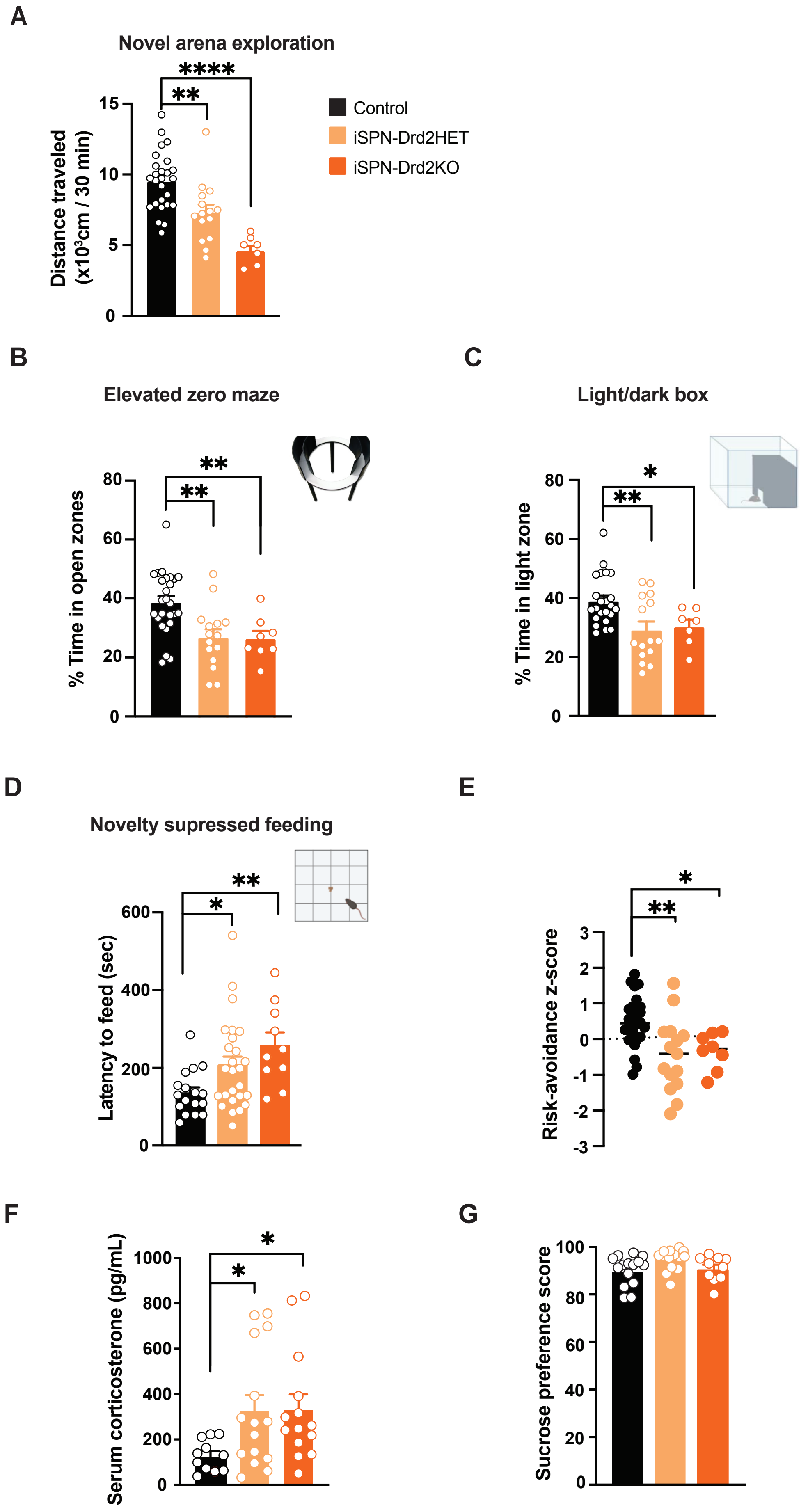

Supplementary Figure 6.

(A) Heterozygous iSPN-Drd2HET and homozygous iSPN-Drd2KO mice both showed reduced locomotion in a novel arena relative to controls in a “gene-dose” manner, with homozygous Drd2 knockout mice showing the most significant reduction in distance traveled ($F(2,46)=20.54$, $p<0.0001$, control vs. iSPN-Drd2HET: $p<0.01$; control vs. iSPN-Drd2KO: $p<0.0001$, iSPN-Drd2HET vs. iSPN-Drd2KO: $p<0.01$, n=7-26/genotype). (B) Both hemizygous and homozygous Drd2 mice show decreased time in the exposed sections of the EZM ($F(2,46)=8.7$, $p<0.001$, control vs. iSPN-Drd2HET: $p<0.001$; control vs. iSPN-Drd2KO: $p<0.01$, 8-26/genotype), and (C) light-dark box ($F(2,46)=6.49$, $p<0.01$, control vs. iSPN-Drd2HET: $p<0.01$, control vs. iSPN-Drd2KO: $p<0.05$, n=8-26/genotype), as well as (D) a latency to feed on the novelty suppressed feeding ($F(2,50)=5.3$, $p<0.01$; control vs. iSPN-Drd2HET: $p<0.05$, control vs. iSPN-Drd2KO: $p<0.01$, n=10-26/genotype). However, unlike the locomotion, no gene dose was observed. (E) Across all tasks, a composite z-score of all behaviors indicated that both iSPN-Drd2HET and KO mice show less exploration of risky zones compared to littermate controls ($F(2,46)=7.86$, $p<0.001$; control vs. iSPN-Drd2HET: $p<0.001$; control vs. iSPN-Drd2KO: $p<0.05$, n=8-16/ genotype). (F) Increased basal serum corticosterone levels were seen in both iSPN-Drd2HET and iSPN-Drd2KO mice compared to littermate controls ($F(2,37)=3.4$, $p<0.05$; control vs. iSPN-Drd2HET: $p<0.05$; control vs. iSPN-Drd2KO: $p<0.05$, n=11-16/genotype). (G) No differences were seen in a sucrose anhedonia test across genotypes ($F(2,34)=1.07$, $p>0.05$, n=10-15/genotype). Data is presented as mean and quartiles or mean ± SEM, *$p<0.05$, **$p<0.01$, ****$p<0.0001$.

Suppl Fig 7 Bocarsly et al.

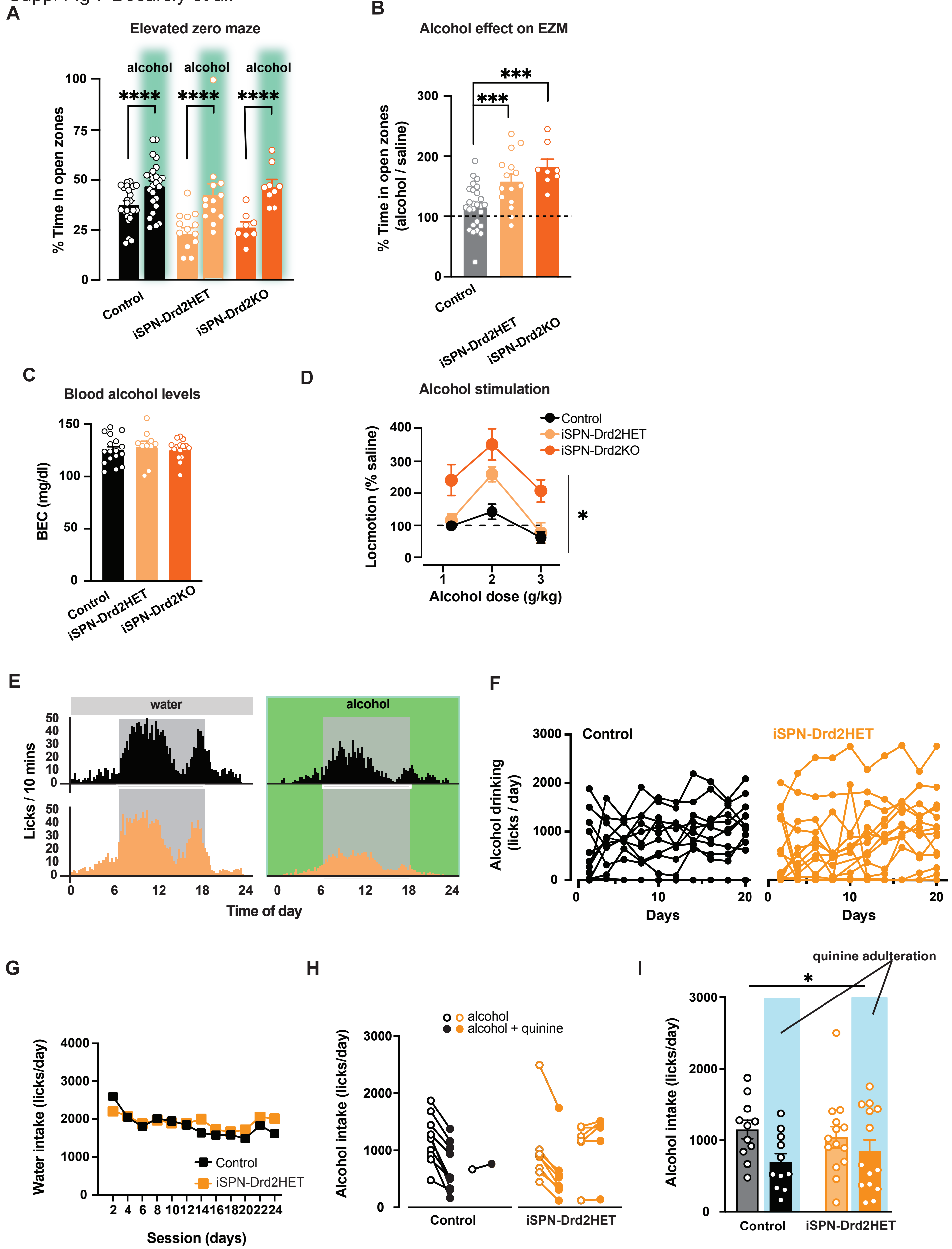

Supplementary Figure 7.

(A) Groups of littermate control, iSPN-Drd2HET, and iSPN-Drd2KO mice were given i.p. injections of either saline or alcohol (1.2 g/kg) and placed on the EZM. For all genotypes, mice spent more time in the open zones following alcohol injection as compared to saline (main effect of drug: $F(1,90)=32.0$, $p<0.0001$; main effect of genotype: $F(2,90)=5.5$, $p<0.01$; control mice saline vs. alcohol: $p<0.0001$. iSPN-Drd2HET saline vs. alcohol: $p<0.0001$. iSPN-Drd2KO saline vs. alcohol: $p<0.0001$, n=8-26/group). (B) When time spent on in the open zones following alcohol was normalized to time spent in the open zones following saline, the iSPN-Drd2HET and iSPN-Drd2KO mice showed a greater change in percent time spent in the open compared to littermate controls, indicating elevated anxiolytic effects ($F(2,45)=13.1$, $p<0.0001$; control versus iSPN-Drd2HET: $p<0.001$; control versus iSPN-Drd2KO: $p<0.001$, n=8-26/genotype). (C) Blood ethanol concentrations (BEC) were no different across genotypes 10 min after i.p. administration of 1.2 g/kg alcohol ($F(2,39)=0.24$, $p>0.05$, n=10-17/genotype). (D) iSPN-Drd2HET and iSPN-Drd2KO mice show a gene ($F(2,33)=27.77$, $p<0.0001$) and dose ($F(2,63)=17.66$, $p<0.0001$) effect on the locomotor response to alcohol, with both iSPN-Drd2HET and iSPN-Drd2KO mice showing increased alcohol locomotion at 2 g/kg compared to littermate controls ($F(2,30)=12.80$, $p<0.0001$). The Intellicage testing system (TSE) was used to group house mice, while still collecting individual data on alcohol intake. (E) Diurnal patterns of licks for both alcohol and water were recorded, showing most of the fluid consumption in the dark cycle (gray shaded area). (F) Each line represents the alcohol lick behavior of an individual mouse. Both iSPN-Drd2HET mice and littermate controls showed individual variability in alcohol licks. (G) Water intake on the days alcohol was concurrently available was no different between iSPN-Drd2HET mice and littermate controls ($F(11,241)=1.07$, $p>0.05$, n=11-14/ genotype). (H) When the alcohol was adulterated with 0.5 mM quinine, most of the control mice decreased their intake, while many iSPN-Drd2HET mice did not. (I) While an overall effect was seen on alcohol intake with 0.5 mM quinine adulteration ($F(1,23)=4.95$, $p<0.05$, n=11-14/genotype), This effect was predominantly driven by a decrease in intake in the control group rather than the iSPN-Drd2HET group. Data presented as individual data points, lines designate within-subject, or mean ± SEM. *$p<0.05$, ***$p<0.001$, ****$p<0.0001$.
